# Aromatized to Find Mates: α-Pinene Aroma Boosts the Mating Success of Adult Olive Fruit Flies

**DOI:** 10.1371/journal.pone.0081336

**Published:** 2013-11-19

**Authors:** Christos D. Gerofotis, Charalampos S. Ioannou, Nikos T. Papadopoulos

**Affiliations:** Laboratory of Entomology and Agricultural Zoology, Department of Agriculture Crop Production and Rural Environment, University of Thessaly, Volos, Greece; University of Crete, Greece

## Abstract

**Background:**

Contrary to other Tephritidae, female but also male olive flies, *Bactrocera oleae* release pheromones during their sexual communication. Alpha-pinene, a common plant volatile found in high amounts in unripe olive fruit and leaves has been detected as one of the major components of the female pheromone. However, possible effects of α-pinene and that of other host volatiles on the mating behavior of the olive fly have not been investigated.

**Methodology:**

Using wild olive flies, reared on olive fruit for 3 generations in the laboratory, we explored whether exposure of male and female olive flies to α-pinene affects their sexual performance.

**Results:**

Exposure of sexually mature adult olive flies to the aroma of α-pinene significantly increases the mating performance over non-exposed individuals. Interestingly, exposure to α*-*pinene boosts the mating success of both males and female olive flies.

**Conclusions:**

This is the first report of such an effect on the olive fly, and the first time that a single plant volatile has been reported to induce such a phenomenon on both sexes of a single species. We discuss the possible associated mechanism and provide some practical implications.

## Introduction

Plant volatiles are of strong ecological importance shaping behavioural and physiological responses in insects [1]. They provide important cues to insect species for locating food sources [2–4], finding suitable oviposition sites [5,6] or mediating oviposition behavior [7,8]. Besides, there have been many studies demonstrating that volatiles of host and non-host plants may exert a profound effect on the sexual behavior of insects [9] by facilitating mate finding [10–12] or modifying mate selection strategies (i.e. sexual signaling, courting) [9,10]. Furthermore, the feeding activity of herbivores may result in a distinct blend of plant volatiles that (a) may facilitate colonization by attracting more herbivores [13,14] or (b) on the other hand, may be used by carnivorous insects (predators and parasitoids) to detect prey or hosts [15–17]. 

Over the last two decades, much information has been gathered regarding effects of plant volatiles on the adult sexual behavior of several species of the family Tephritidae (true fruit flies), which includes several of the most important insect pests of agricultural commodities worldwide [18,19]. In certain species, exposure of males to particular plant compounds of host or non-host species (usually a blend of essential oils) confers a mating advantage over individuals denied access to such substances [19–23]. Mating-enhancing chemicals usually elicit strong attraction for males, who have been frequently reported to feed on the source of the odor [19,24–27]. In one of the most thoroughly studied systems, males of the oriental fruit fly, *Bactrocera dorsalis*, are strongly attracted to methyl eugenol, which is ingested and used as precursor in sex pheromone synthesis [27]. Hence, males exposed to methyl eugenol become more attractive to females searching for mates than methy eugenol-deprived ones and, thereby, more successful in reproduction [20]. Furthermore, males of the Mediterranean fruit fly (medfly), *Ceratitis capitata* acquire a significant mating advantage after being exposed to ginger root oil, citrus oils and to synthetic attractants such as trimedlure [21,22,28]. Alpha-copaene, a sesquiterpene contained in ginger root oil and citrus oils, as well as other compounds seems to be involved in enhancing the mating performance (copulatory success) of male medflies [28–30]. Likewise, exposure of males of the south American fruit fly, *Anastrepha fraterculus* to guava fruit volatiles enhance their sexual performance [31]. The wealth of information regarding effects of plant volatiles on fruit fly behaviors has been taken also into practical use in the case of the Mediterranean fruit fly. Today thanks to work of Shelly and colleagues [32,33] in most Sterile Insect Release programs against the Mediterranean fruit fly sterilized males are massively exposed to ginger root oil aroma before being released [34].

**Figure 1:**
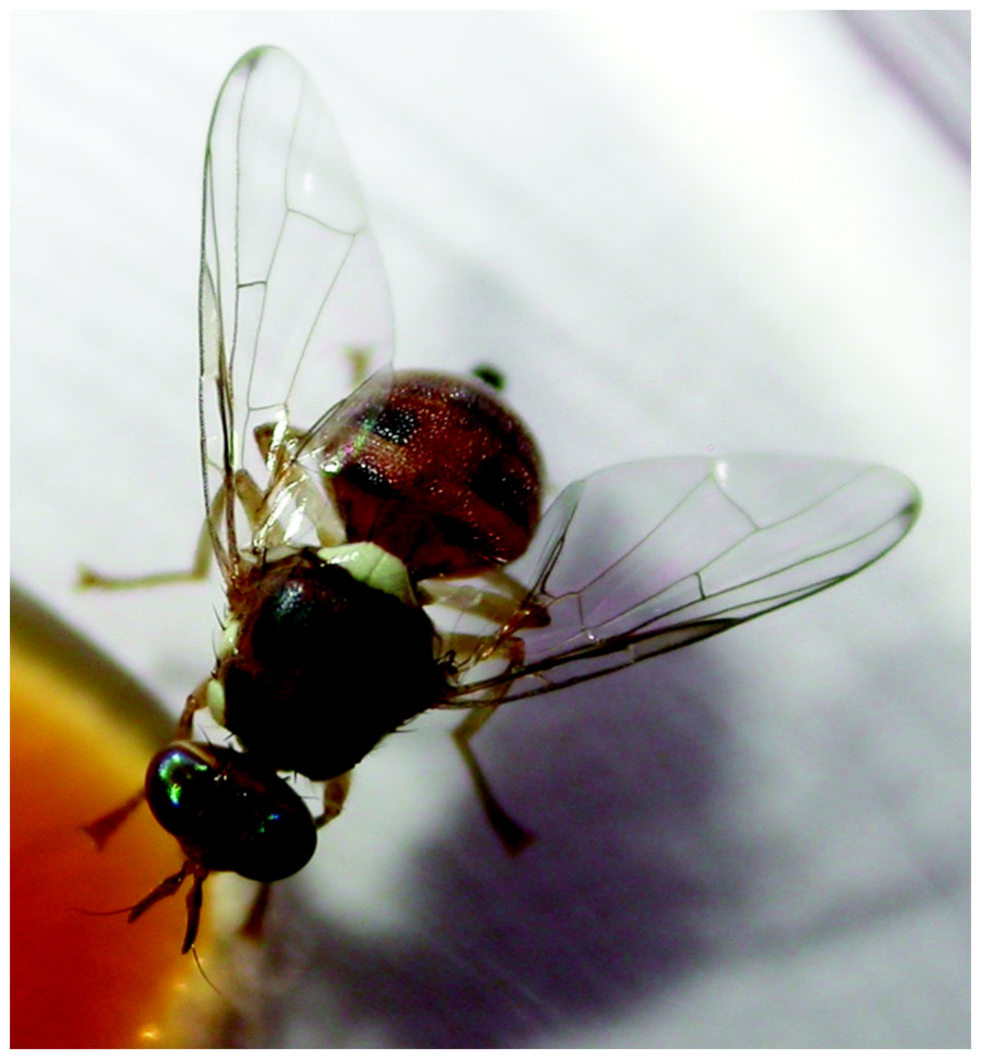
Female olive fly.

The olive fly (Figure 1) is a monophagous, multivoltine species that following its recent invasion in North America (California, North Mexico) [35–37] now threatens almost the entire olive producing industry of the world and causes an enormous economic loss for olive growers each year. Despite decades of intensive research [38–40] the mating system of the olive fly has not yet been resolved in detail. Males are polygynous, while a proportion of females may mate more than once during the life course [41]. Similar to other species of the genus mating takes place at dusk [42]. However, in contrast to other tephritids, it is the female that releases a sexual pheromone in this species, although a few studies report that males release smaller quantities of the same sexual pheromone as well [43,44]. Recent re-assessment of the control of the olive fruit fly using the Sterile Insect Technique [45] has led to a resurgence of interest in the mating behavior of this species, which has been extensively studied in the past [38,39,46]. Laboratory trials reveal three distinct phases that lead to successful copulation (a) mate search or the attraction of males by females [43], (b) courtship or the short range assessment of male quality by females based on male wing vibration and possible other traits, and (c) copulation attempts and tactile stimuli during mounting that may be used by females to further assess males and lead to successful insemination [47] (and references therein). 

Female olive flies release a sexual pheromone attractive to males that contains four components: (a) olean (1,7-dioxaspiro[5.5]-undecane), (b) methyl dodecanoate, (c) α-pinene, and (d) nonanal [48]. Interestingly, male olive flies are also able to attract virgin females [49] and body extracts of olive fly males are attractive to virgin females [50]. Young males have been reported to release olean (the main component of the female sexual pheromone) [51,52]. More recent studies have demonstrated that sexually mature males in addition to olean also produce muscalure ([Z]-9-tricosene), a male specific pheromone that selectively attracts females [43]. It was also recently demonstrated that the production of olean by male olive flies does not affect their mating success, and the hypothesis that the production of olean is a form of female chemical mimicry has been rejected [47]. Apparently, despite the wealth of older studies and the significant progress achieved over the last few years, the role and function of pheromonal signals, as well as details of sexual communication in wild olive flies remain unknown. 

Similar to other insect species [53,54] (for review see ‘[55]’), the female pheromone blend of the olive fly contains the plant compounds α-pinene and nonanal, both of which exhibit a synergistic action with olean (the main component of the pheromone) in attracting males [56]. Neither nonanal (an oxidation byproduct) nor α-pinene are known to be synthesized de novo in insects, and they are most probably acquired by plant materials. Alpha-pinene is one of the most common volatiles in nature, released by a wide range of plant species, including coniferous trees, rosemary, lavender, turpentine and olive [57]. There are many properties, such as anti-inflammatory, fungicidal, bactericidal, and insecticidal that have rendered α-pinene a chemical of general use [57]. In insects it has been shown that α-pinene may have a synergistic or inhibitory action with aggregation pheromones on the attraction of bark beetles to host plants [58,59], and it has been used to enhance the attractiveness of trap baits used in pest management programs [60]. Although α-pinene by itself does not attract predators, it enhances significantly the attraction of some predators to the pheromone of their corresponding prey [61]. On the other hand, α-pinene serves as pheromone precursor in pheromone biosynthesis of scolitids [62,63]. Alpha-pinene was also isolated in the frontal gland secretion of soldier volatile releases in *Reticulitermes* species [64]. 

As pointed out earlier, α-pinene (emitted at high rates by semi-ripe olive fruit and leaves) is one of the four major compounds of female sexual pheromone [48] that increases male attraction to synthetic pheromone [44], stimulates oviposition [65] and evokes electrophysiological responses in both male and female antennae [66]. Nonetheless, the effects of α-pinene on the mating success of adult olive flies have not yet been addressed. 

Working towards understanding the complex mating system of the olive fly and the role of olive tree volatiles on their sexual behavior, and as much as α-pinene is an important component of the olive fly reproductive behavior, synergistically increasing the attractiveness of the female sexual pheromone, here for the first time we provide solid evidence that a single volatile (α-pinene) from the host plant (the olive fruit) boosts the mating competitiveness of both sexes of the olive fly.

## Materials and Methods

### Ethics statement

This study was conducted in the laboratory using flies collected from the wild. There is no specific permission required for collecting wild olive flies, since this is a major pest of olives in the area and is, therefore, neither an endangered nor a protected species.

The experiments were conducted at 24 ± 1°C, 60 ± 5% R.H, and a photoperiod of L14: D10, with photophase starting at 07:00, in the laboratory of Entomology and Agricultural Zoology at the University of Thessaly, Greece during autumn - spring 2011 - 2012. In all experiments, the flies used originated from field-infested olives, collected in Volos in August 2011, and were reared for 1 to 3 generations (F_1_ - F_3_) on olive fruit that had been kept in a storeroom for several months. All adults were maintained in wooden nylon-screened holding cages (30 x 30 x 30 cm) and had free access to food [mixture of yeast hydrolysate (MP Biomedicals LLC., France) and sugar in a ratio of 1:4] and water. Olive fruit were offered to females for oviposition. Therefore, larval development took place in olive fruit, and the resulting pupae were kept under the same conditions noted above. 

### Effect of α-pinene on males

Flies were sexed within 24 h of emergence. Females were introduced individually into small cages made of transparent drinking cups (400-ml volume) with an opening 3x8 cm covered with nylon mesh to allow adequate ventilation. Ample adult food and water were provided in each cage. Males were kept in groups of 40 in plexiglass cages (20 cm cubes with a cloth sleeve on two sides for ventilation) with food and water. On day 10 of their adult life, to distinguish between treated and control individuals, males of each category were marked on the pronotum with a small dot of water-based paint of different colors after being anesthetized with CO2. Four days following color marking, 10 males of each category were transferred into small cubical plexiglass cages (15 cm per side with a round opening 7.5 cm in diameter on one side) and exposed to 5, 10, 20, 40 and 80 μl of (-)-*α*-pinene (98% purity, Alfa, Aesar, France) or water for the control. Alpha-pinene or water was applied onto a disk of double layer white filter paper placed on the bottom of a cylindrical plastic cup (3 cm diameter, 1 cm height). To control evaporation, and prohibit direct contact, each cup was placed in a 5.5-cm diameter plastic Petri dish provided with 5 ml of distilled water. The cover of the dish bore a 5-cm diameter hole in which a plastic, hollow, red hemisphere (hereafter called dome) of the same diameter was fitted. The dome had 100 equidistant holes (≈0.5 mm diameter) through which the substance was evaporating. Each dome was placed inside the exposure cages for 3 successive days before performing the mating tests. At 10:00h on the next two days an additional amount of α-pinene depending on trial (5, 10, 20, 40 and 80 μl) was added on the same filter paper. Following application of α-pinene there was an increase in locomotory activity of males observed. 

At 10:00h of the experimental day both treatment and control domes were removed from male cages. Two males (one exposed and one non-exposed) were introduced into each individual cage housing a virgin female at 17:30h. Therefore, each replicate consisted of one treated male competing with one untreated male for a virgin female in the screen-covered, transparent plastic cup. Mating success of each male category, as well as latency to mate time, and copula duration were recorded. Both males and females used in the above tests were sexually mature virgins (17 days old). We ran 100 replicates for each different dose of exposure.

### Effect of α-pinene on females

Upon emergence, groups of 40 females were placed in plexiglass cages (20 cm cubes with a cloth sleeve on two sides). To distinguish between treated and control individuals, on day 10 of their adult life females of each category were marked, as described above for males. Four days following color marking, groups of 10 females of each category were transferred into 15 cm plexiglass cages. Female exposure to α-pinene was conducted following the same procedure as described for the males, though using a single dose of 20 μl (chosen because it induced the highest response among males, see below). Upon emergence, males were introduced individually to 400-ml volume cages (see above). Procedures on the testing date were identical with those given earlier for males. In fact, one exposed and one non-exposed to α-pinene female were introduced into a male’s cage and were competing for mating. Both males and females used were sexually mature 17 days old. The percentage of matings, the latency to mate, and the copula duration were recorded. We conducted 100 replicates. 

### Statistical analyses

Departure from random mating was assessed using the chi-square test. An independent t-test was applied to detect differences in mating duration and latency to mate between exposed and non-exposed individuals. 

## Results

Reproductively mature males of the olive fly that were exposed to pure α*-*pinene gained a significant, dose-dependent mating advantage over non exposed males (Table 1). A significant difference between treated and control males was observed for the doses of 10 and 20 μl, respectively, with peak advantage reported at 20 μl. At this dose, exposed males achieved 82% of all matings. Higher doses seemed to induce some negative effects (though not significant), while lower ones failed to reach significance. 

**Table 1:** Mating rates of olive fruit fly adults exposed to α-pinene (treatment) or maintained non exposed to any compound (control).

				**Proportion of matings**		
		**α-pinene exposure dose (μl)**	**N**	**Treatment**	**Control**	***P***
**Sex**	Males	5	81 (100)	0.59	0.41	0.094
		10	83 (100)	0.61	0.39	0.036*
		20	84 (100)	0.82	0.18	<0.001*
		40	82 (100)	0.46	0.54	0.500
		80	78 (100)	0.42	0.58	0.170
	Females	20	98 (100)	0.62	0.38	0.015*

N= number of matings recorded (total number of replicates). Asterisks indicate significance at 0.05 level (Chi-square test).

One of the most striking results that we report here regards the effect of α-pinene on female olive flies. Exposure of reproductively mature, non-mated females to a single dose (20 μl) of α-pinene significantly boosted their mating success (Table 1). 

**Figure 2:**
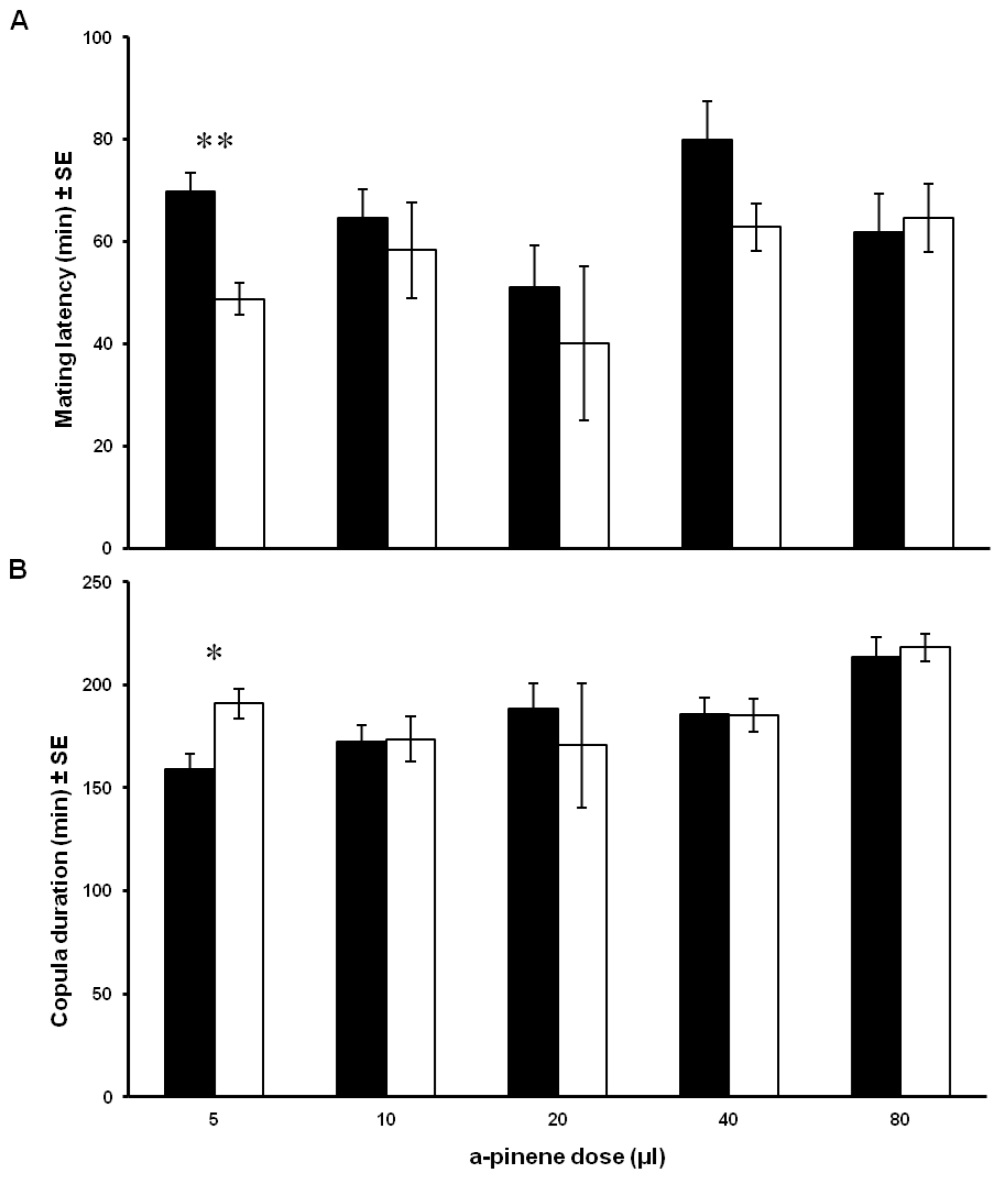
Effect of different α-pinene doses on male olive fruit fly copulation parameters. (**A**) Latency to mate and (**B**) copula duration. Solid and open bars stand for exposed males to α-pinene and non-exposed males respectively. * *P*< 0.05, ** *P*< 0.001 *t*-test, *N*=31-84.

Overall it seems that latency to mate and copula duration was similar for exposed and non-exposed males (paired t-test, P > 0.05). Significant differences were reported at the lower dose of 5 μl only (Figure 2). Likewise exposure of females to 20 μl of α-pinene did not affect the latency to mate or the copulation duration (paired t-test P > 0.05; data not shown, Figure S1). 

## Discussion

Our results demonstrate that exposure to α-pinene, a plant volatile and one of the four components of female sexual pheromone, increases the mating propensity (readiness to mate) of adult olive flies. To our knowledge, this represents the first demonstration that a single plant volatile increases the mating propensity of both sexes in an insect species. It seems that the induced phenomenon is dose dependent at least for males (no such tests were conducted for females). Although there are several cases where plant volatiles and even a single compound increases mating success in other fruit flies of the family Tephritidae there are no reports on effects regarding females [19,21]. In one of the few trials, exposure of female medflies to ginger root oil has not affected their mating propensity [67]. Similar results have been recently obtained from our group when female medflies were exposed to orange oil (Papadopoulos et al. unpublished).

Host plant chemicals may affect the sex pheromone biology of phytophagous insects by acting on both the pheromone releasers and those detecting and responding to the pheromone emissions [9]. As noted earlier, males of several species of fruit flies exposed to plant volatiles acquire a significant mating advantage over non-exposed males. Males of tropical *Bactrocera* species, such as *B. dorsalis, B. cucurbitae* and *B. tryoni* are strongly attracted to and feed on plant or synthetic compounds, such as methyl-eugenol, cuelure or raspberry ketone. Feeding on these compounds elicits a significant mating advantage over males that have no access to these compounds [68,69]. After ingestion, these compounds are biochemically modified and then sequestered in the rectal gland and later emitted as pheromone components [70,71]. In other fruit flies, exposure of males to the aroma of plant derived or synthetic compound elicits similar phenomena and the function of these volatiles is yet to be clarified [22,28,72,73]. For example, regardless of intensive efforts, the role of citrus oils and ginger root oil in increasing the mating performance of Mediterranean fruit fly males has not been elucidated in detail. Both ginger and citrus oils increase male sexual signaling, and pheromone produced from males exposed to citrus oils induces some arrestment effects on virgin females [22]. On the other hand, volatiles may be absorbed by the cuticle of exposed males, and serve as short range stimuli during courtship [74]. Apparently the mechanisms operating in increasing the mating performance of both female and male olive flies remain unknown. There are several facets that make this system challenging to explore, since contrary to (a) other tephritids, both female and male olive flies produce sexual attractants, (b) other *Bactrocera* species, feeding is not required, and (c) all other known cases, exposure to α-pinene increases the mating performance of both males and females.

Exposure of female olive flies to α-pinene may alter the quality and even increase the quantity of the produced pheromone. Alpha-pinene vapors might be absorbed by female exoskeleton as in some scolitid bettles [63] and reach the pheromone producing glands conferring changes on sex pheromone composition rendering the produced blend more attractive for males via synergistic action with olean and the other components of the sexual pheromone [44]. Whether exposure of females to α-pinene increases mating propensity (readiness to mate) or attractiveness to males needs to be investigated in future studies. Additionally, as in the case of other *Bactocera* species [19] (and references therein) [70,75] the acquired α-pinene may serve as precursor for synthesizing other active pheromonal compounds. Although α-pinene has been detected in the pheromone blend of female olive flies, it is not yet known whether females can synthesize it de novo or if they use ingested or absorbed compounds [76]. On the other hand, absorbed quantities of α-pinene may alter the “cuticular smell” of females and serve as a short-range stimulus to courting males [74]. This may involve a male choice for females [77,78]. Finally, female exposure to compounds, such as α-pinene, that indicate the presence of host fruit in an appropriate maturation stage (green olives) and stimulates oviposition [65] could activate hormonal mechanisms associated with reproductive activities and, therefore, increased mating receptivity. 

Similar to females, male exposure to α-pinene could result in increased production or release of muscalure. Alternatively, α-pinene may synergistically act with male pheromone emissions rendering the produced blend more attractive. Likewise, absorption of α-pinene by male cuticle may alter the scent of males and affect female decision to mate during courtship. Exposure to α-pinene may also affect male courtship behavior. 

As (a) this is the first report of increased mating performance of adult olive flies exposed to plant volatiles, specifically to α-pinene, (b) the mating system of the olive fly has yet to be resolved in detail, and (c) the induced phenomenon involves both sexes, it is rather premature to open a discussion on ultimate underlying factors. Whether direct or indirect effects on counterparts mated with adults exposed to α-pinene exist, needs to be addressed in future studies in order to form a solid ground for discussion involving sexual or other forms of selection. Some ideas that could be considered in such a discussion are: first, the scent of α-pinene (absorbed by exoskeleton) of exposed males may indicate a superior ability to locate key compounds associated with critical insect-plant interactions like host location, acceptance and oviposition. Second, female preference for exposed males could represent a “sensory trap” [79] or “sensory exploitation” [80] where the olfactory cues of exposed males exploits a preexisting bias in females that evolved in a different context, such as searching for food or oviposition resources. Third, a series of studies suggest that in some *Bactrocera* species plant chemicals (methyl eugenol) that enhance males’ mating competitiveness may also confer defense benefits against vertebrate predators (i.e. allomonal function) such as lizards and birds [75,81,82]. Given that pairs during copulation are usually immobile and hence highly vulnerable to predation, it would be beneficially adaptive for females to select males that have acquired these chemicals in order to minimize predation risk. Despite the fact that this theory lacks experimental evidence in a natural setting [68], it provides useful fields for further investigations regarding sexual selection in *Bactrocera* species and the olive fly considering the new findings presented here. Apparently, similar hypothesis should be put forward considering females as the beneficiaries of exposure to α-pinene; however, the literature is “male” dominated. 

The association between α-pinene and the male mating performance of this species may be put into practical use for the control of *B. oleae*. The Sterile Insect Technique (SIT) or similar methods, such as the Incompatible Insect Technique (IIT), rely on the mating competitiveness of field released, mass-reared, sterilized males (either through irradiation or transinfection with the endosymbiont *Wolbachia pipientis*) against feral males. Females that copulate with such males lay eggs that do not develop into larvae and, therefore, the target population is suppressed. Hence, large-scale, pre-release treatment of sterilized males with α-pinene may increase their mating performance and thus enhance the effectiveness of both techniques.

Moreover, host plant influences on sex pheromone behavior of phytophagous insects might be of great importance in developing of sex-specific lures. In cases where host plants provide insects with biologically active compounds that interfere and/or influence in their sexual communication, man-made lures based solely on pheromone may not be effective when placed in the wild [9]. In light of our findings, it might be useful to re-examine the synergistic action between *a*-pinene and the commercial available female pheromone component 1,7-dioxaspiro[5.5] undecane (olean) in order to improve male attraction. 

Besides practical implications, our findings shed important light on the mating behavior of the olive fly and pose a list of proximate and ultimate questions that need to be tested in the near future. The complex mating system of the olive fly where both sexes seem to release inter- and intrasexual attractants provides a challenging ground for further research. Current progress on pheromonal stimuli emitted by male and female olive flies together with our findings may help understand the olfactory communication between the sexes of the olive fly and the involvement of plant volatiles, such as α-pinene.

## Supporting Information

Figure S1 **Effect of α-pinene on female olive fruit fly copulation parameters.** Latency to mate and copula duration. Solid and open bars stand for exposed females to 20 μl α-pinene and non-exposed females respectively. *N*=98.
(PPTX)
