# Supplementary figures and images for: Aromatized to Find Mates: α-Pinene Aroma Boosts the Mating Success of Adult Olive Fruit Flies
Source: PLoS One. 2013 Nov 19;8(11):e81336. doi: 10.1371/journal.pone.0081336 (PMC3834339; doi:10.1371/journal.pone.0081336)

## Slide 1
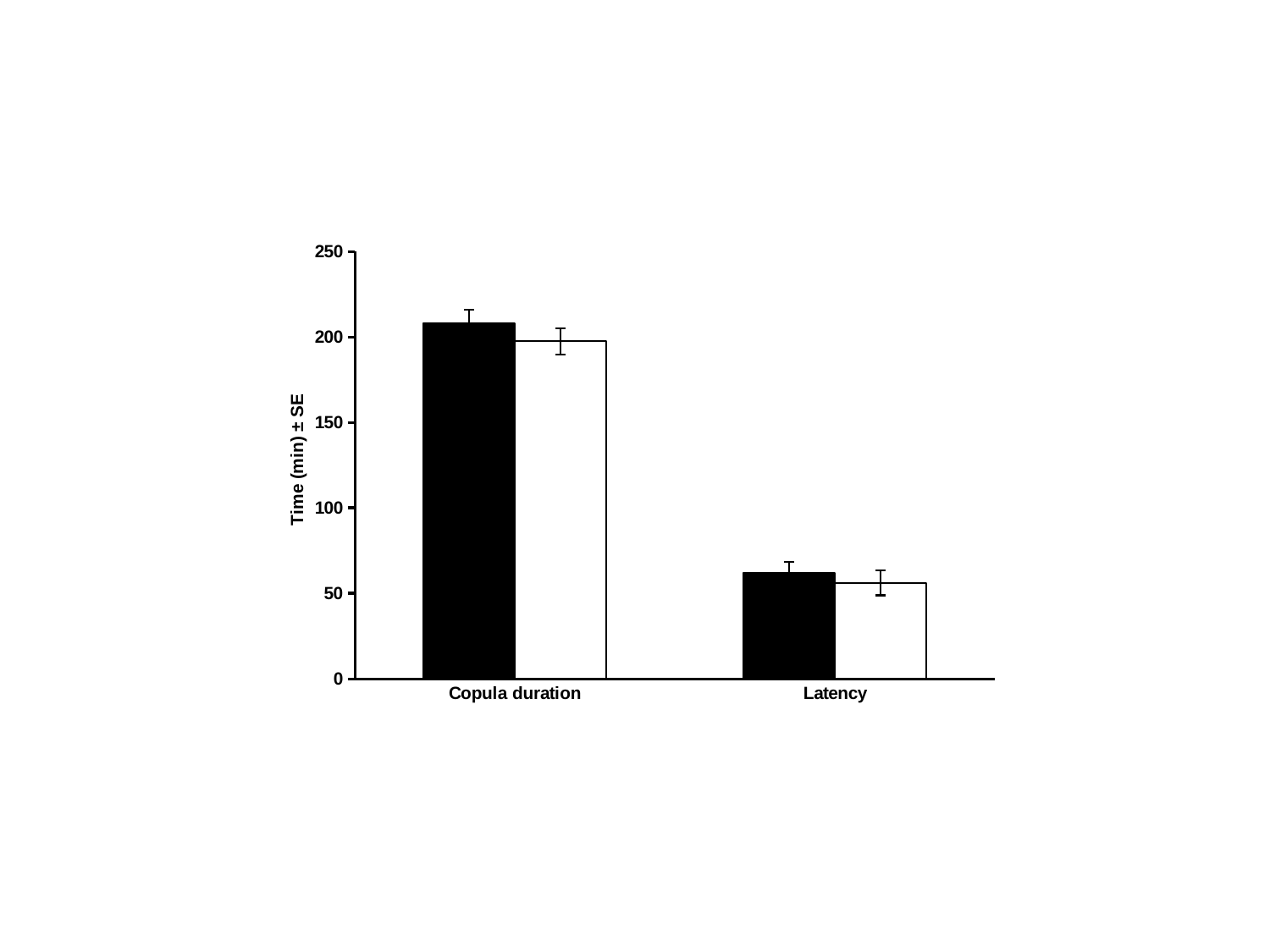

### Chart
| Category | FEMALES Treatment | FEMALES Control |
|---|---|---|
| Copula duration | 208.01639344262296 | 197.43243243243242 |
| Latency | 61.9672131147541 | 56.08108108108111 |

Supplement: Figure S1 — Effect of α-pinene on female olive fruit fly copulation parameters. Latency to mate and copula duration. Solid and open bars stand for exposed females to 20 μl α-pinene and non-exposed females respectively. N=98. (PPTX) [file pone.0081336.s001.pptx]
